# Supplementary material for: Data-driven grading of acute graft-versus-host disease
Source: Nat Commun. 2023 Nov 28;14:7799. doi: 10.1038/s41467-023-43372-2 (PMC10684603; doi:10.1038/s41467-023-43372-2)
Supplement: Supplementary file 1 — Supplementary Information [file 41467_2023_43372_MOESM1_ESM.pdf]

Supplementary Information to

Data-driven grading of acute graft-versus-host disease

Evren Bayraktar ǂ[1, 2, 3], Theresa Graf ǂ[1,2], Francis A. Ayuk[4], Gernot Beutel[5], Olaf Penack[6], Thomas Luft[7], Nicole Brueder[5], Gastone Castellani[8], H. Christian Reinhardt[2, 9, 10], Nicolaus Kröger[4], Dietrich W. Beelen[2, 9] and Amin T. Turki* [1, 2, 9, 10, 11, 12]

ǂ These authors contributed equally.

Affiliations:

1 Computational Hematology Lab, West-German Cancer Center, University Hospital Essen, Hufelandstr. 55, 45122 Essen, Germany

2 Department of Hematology and Stem Cell Transplantation, West-German Cancer Center, University Hospital Essen, Hufelandstr. 55, 45122 Essen, Germany

3 Chair III of Applied Mathematics, TU Dortmund University of Applied Sciences, Vogelpothsweg 87, 44227 Dortmund, Germany

4 Department for Stem Cell Transplantation, Medical Center Hamburg-Eppendorf (UKE), Martinistraße 52, 20251 Hamburg, Germany

5 Department of Hematology, Hemostasis, Oncology and Stem Cell Transplantation. Hannover Medical School, Carl-Neuberg-Str. 1, 30625 Hannover, Germany

6 Charité – Universitätsmedizin Berlin, corporate member of Freie Universität Berlin and Humboldt-Universität zu Berlin, Department of Hematology, Oncology and Tumorimmunology, Augustenburger Platz 1, 13353 Berlin, Germany

7 Department of Internal Medicine V, University Hospital Heidelberg, Im Neuenheimer Feld 410, 69120 Heidelberg, Germany

8 Department of Applied Physics and Biophysics, Experimental, Diagnostic and Specialty Medicine - DIMES, University of Bologna, Via Zamboni 33, 40126 Bologna, Italy

9 German Cancer Consortium (DKTK), Partner sites Essen/Düsseldorf, Hufelandstr. 55, 45122 Essen, Germany.

10 Cancer Research Center Cologne Essen (CCCE), Partner site Essen, Hufelandstr. 55, 45122 Essen, Germany

11 Department of Hematology and Oncology, Marienhospital University Hospital, Ruhr-University Bochum, Universitätsstr. 150, 44801 Bochum, Germany

12 Institute for Experimental Cellular Therapy, University Hospital Essen, Hufelandstr. 55, 45122 Essen, Germany

*** Corresponding author:** Priv.-Doz. Dr. med. Dr. phil. Amin T. Turki, MD PhD, Computational Hematology Lab, West-German Cancer Center, University Hospital Essen, University of Duisburg-Essen, Hufelandstrasse 55, 45122 Essen; email: amin.turki@uk-essen.de

Supplementary Information – Table of Contents

Supplementary Figures page 3

Supplementary Tables page 18

Supplementary Methods page 20

Supplementary Notes page 23

Supplementary References page 26

Supplementary Figures

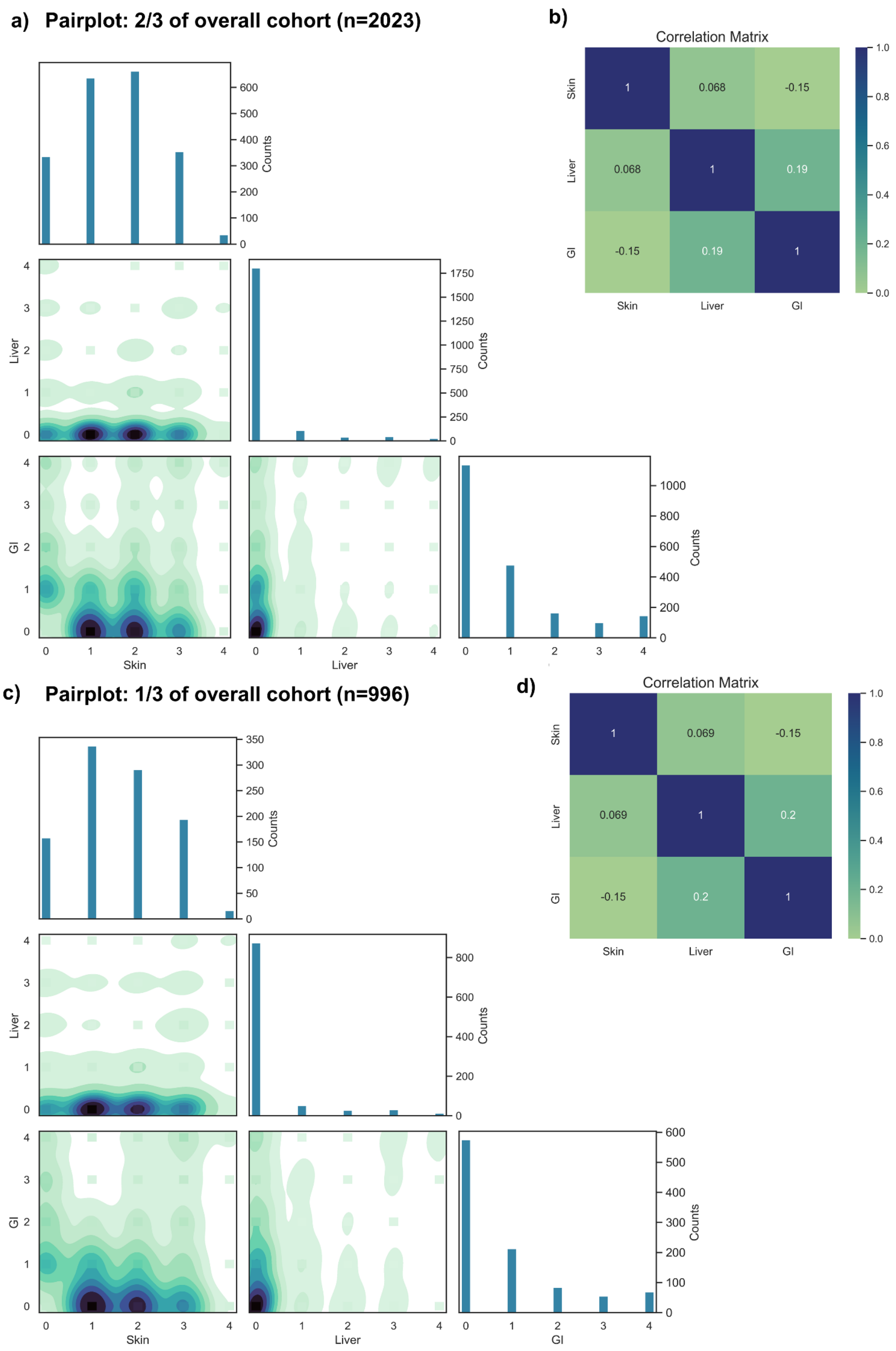

Supplementary Figure 1. Exploratory data analysis of the dataset using a ⅔ – ⅓ split

a Pair plot analysis of ⅔ split cohort (n=2023) plotting the clinical target organ stages of skin, liver and GI involvement (stages 0-4, left to box and below). The target organ stage correlations

are presented as density plots. Patient numbers (*n*) of each subgroup are indicated right to each box. Higher *n* in each subgroup is shown by greater surface coverage. Density of aGVHD target organ combinations is indicated from light green to dark blue. **b** Target organ stage correlation matrix (Spearman) of the training cohort shows the distribution of single variables skin, liver and GI and their respective interactions. Range from -1.0 to +1.0, dark blue indicates full overlap. **c** Pair plot and **d** Target organ stage correlation matrix (Spearman) of ⅓ split cohort (n=996). Analysis, labels and colors as in **a-b**.

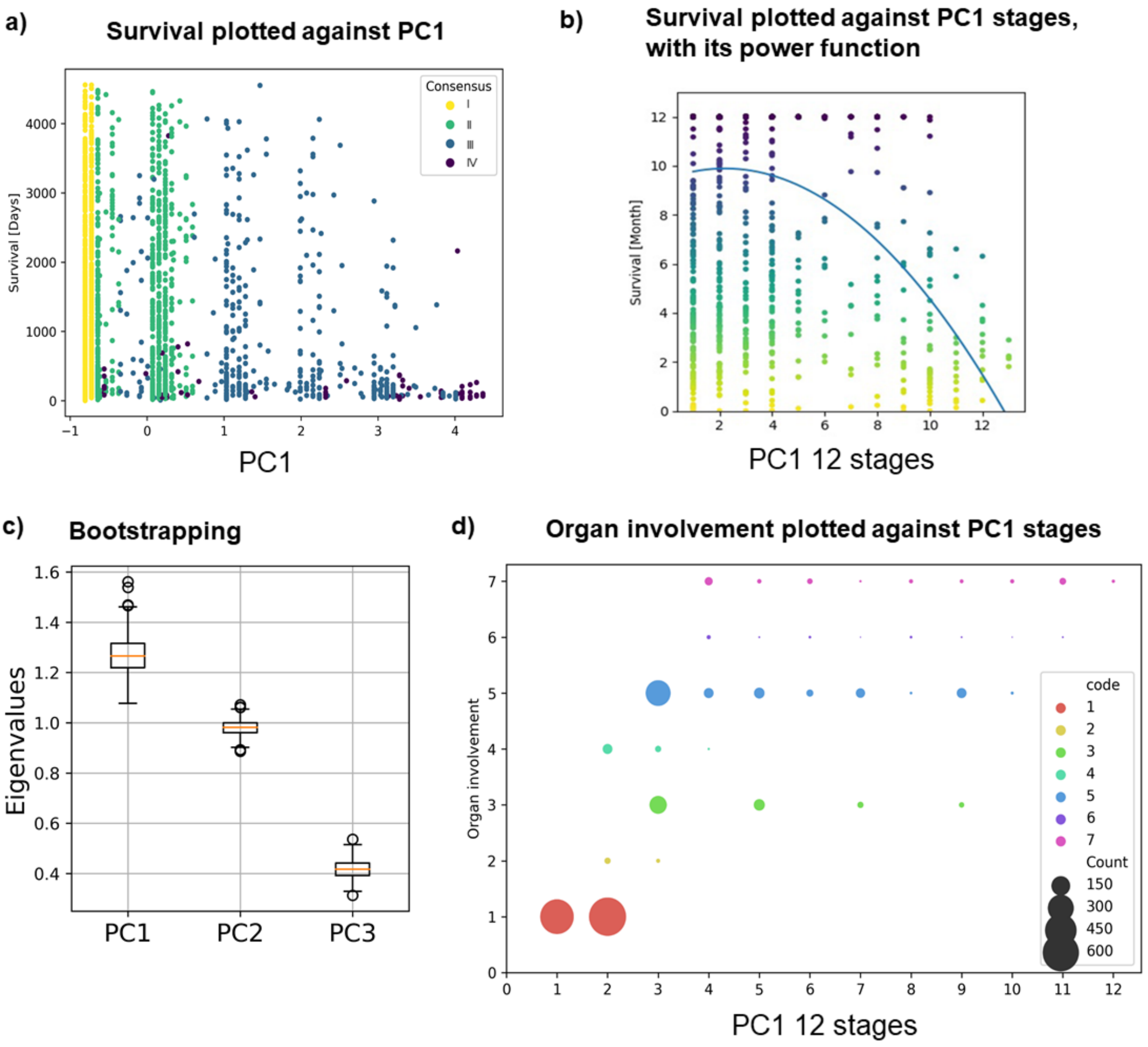

Supplementary Figure 2. Internal Validation and additional characteristics of PC1-based grading development, training cohort (n=2319)

a Plotting of PC1 and overall survival (OS, days from HCT without censoring) reveals lower OS with increasing PC1. Each dot representing one patient with aGVHD. Colored dots representing Consensus grade I-IV (I=yellow, II=green, III=blue, IV=violet) indicate overlap between Consensus grades I & II and II & III on the PC1 axis. **b** Plotting of PC1 stages and OS (months from HCT, censoring < 12 months has not been considered in this representation, patients surviving > 12 months were censored at the 12 months dot) with a power function derived from maximal OS confirms lower OS with increasing PC1 within the first 12 months after HCT. Colors as in B. **c** 500-fold bootstrapping of PCA on training cohort sample (each

sample with size n=1546). The boxplot shows the eigenvalues of each PC (median, orange middle line; 25th, 75th percentile, box). The two lines outside of the box represent the 5th and 95th percentile (whiskers). Single points outside the whiskers are outliers. Source data are provided as a source data file. **d** Color-coded plotting of PC1 stages against aGVHD organ involvement (combinations: 1:skin, red, 2: liver, yellow 3: GI, lime 4: skin and liver, green 5: skin and GI, blue 6; liver and GI, violet 7: skin and liver and GI purple). The circle size corresponds to the *n* of patients in each category.

Visualization of different aGVHD grading methods using tSNE, training cohort (n=2319)

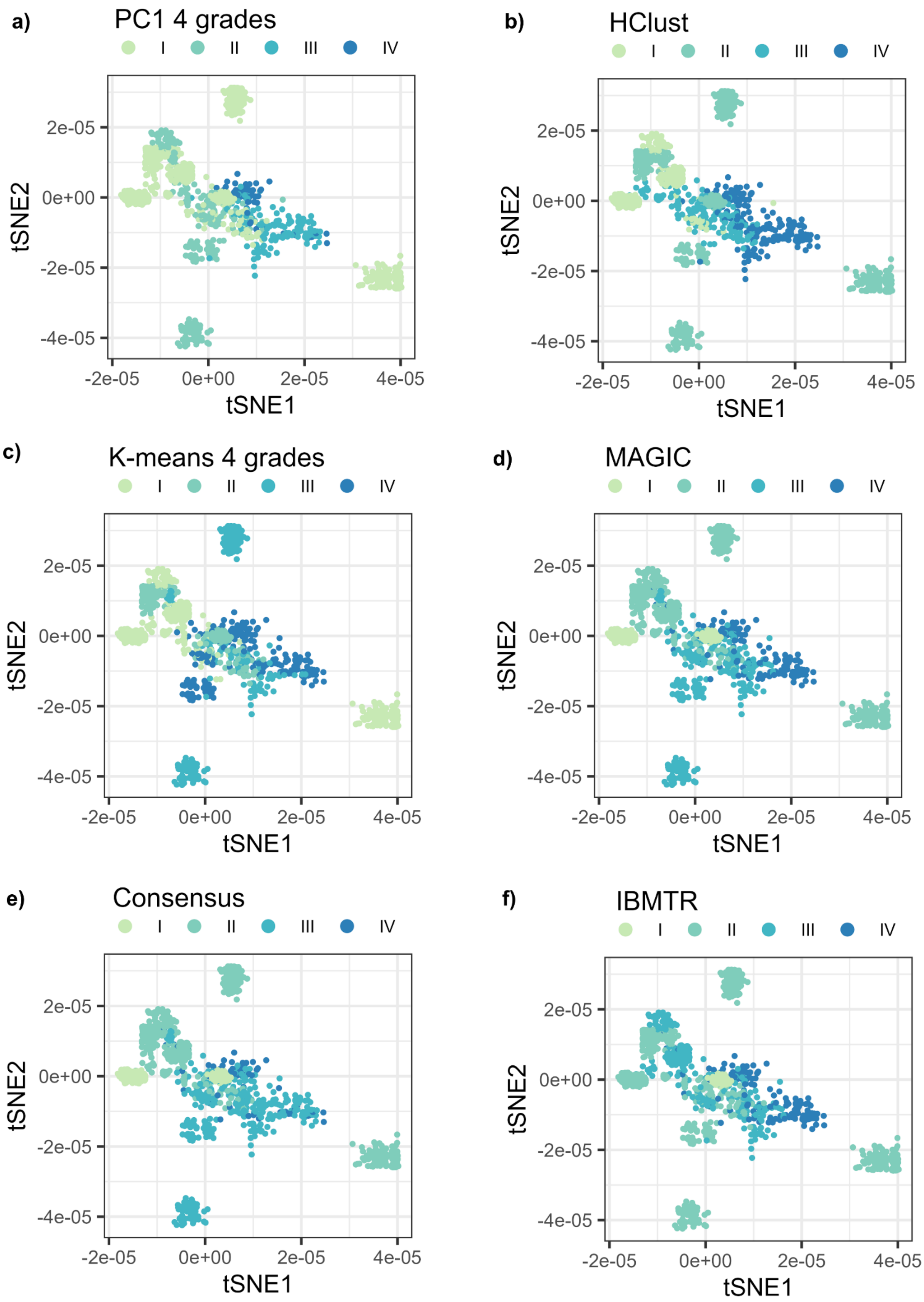

Supplementary Figure 3. Visualization of different aGVHD grading methods using t-SNE, training cohort (n=2319)

t-SNE as nonlinear dimensionality reduction method applied for visualizing grading in the

training cohort (n=2319) on two-dimensional data (tSNE1 and t-SNE2) with each dot representing a single patient. Patients are colored according to their corresponding severity grading (grade I, light green; grade II, dark green; grade III, teal, grade IV, blue). **a** PC1 4 grades, **b** Hierarchical clustering 4 grades, **c** K-means 4 grades. **d** MAGIC 4 grades, **e** Consensus 4 grades, **f** IBMTR 4 grades.

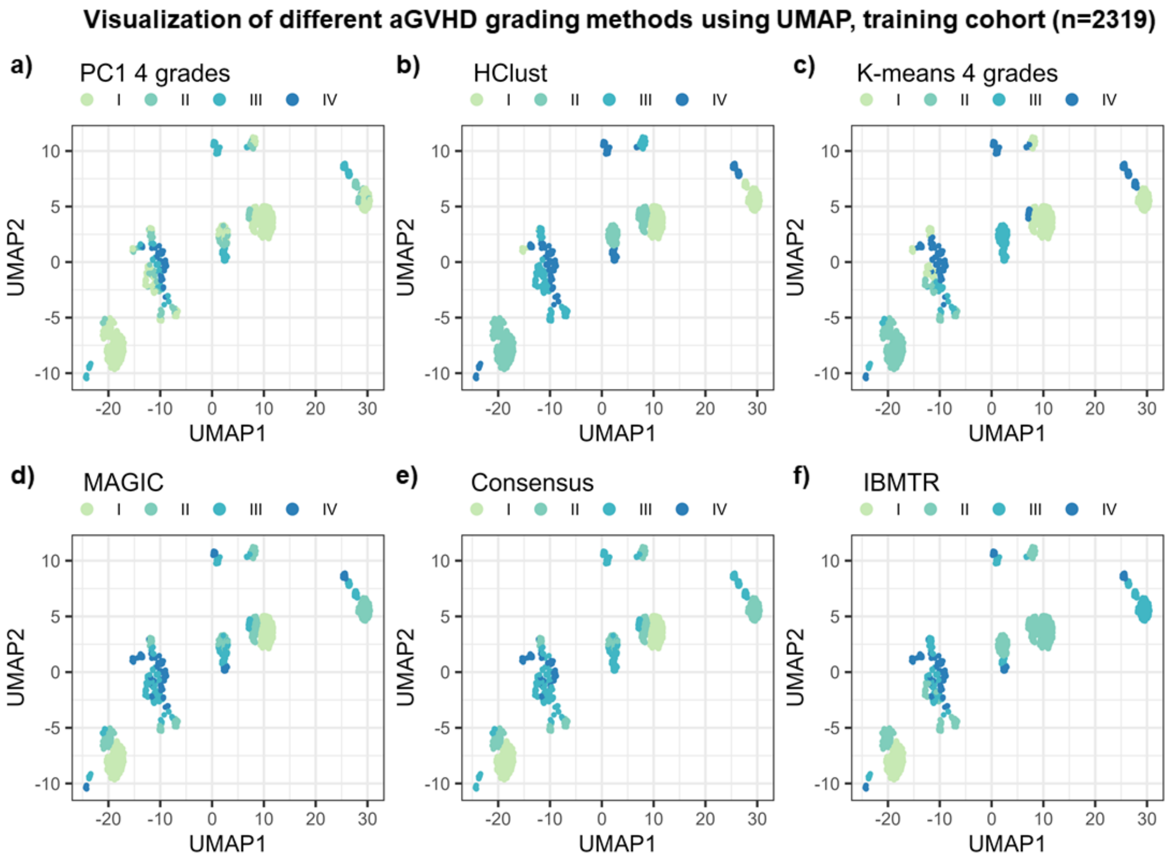

Supplementary Figure 4. Visualization of different aGVHD grading methods using UMAP, training cohort (n=2319)

Uniform Manifold Approximation and Projection (UMAP) as nonlinear dimensionality reduction method was applied for visualizing grading on data in two dimensions (UMAP1 and UMAP2) with each dot representing a single patient. Patients are colored according to their corresponding severity grading (grade I, light green; grade II, dark green; grade III, teal, grade IV, blue). **a-c:** Data driven aGVHD grading methods, **a** PC1 4 grades, **b** Hierarchical clustering 4 grades, **c** K-means 4 grades. **d-f**: Conventional aGVHD grading methods, **d** MAGIC 4 grades, **e** Consensus 4 grades, **f** IBMTR 4 grades.

Visualization of ascending DBSCAN cluster numbers on UMAP output and OS association, training cohort (n=2319)

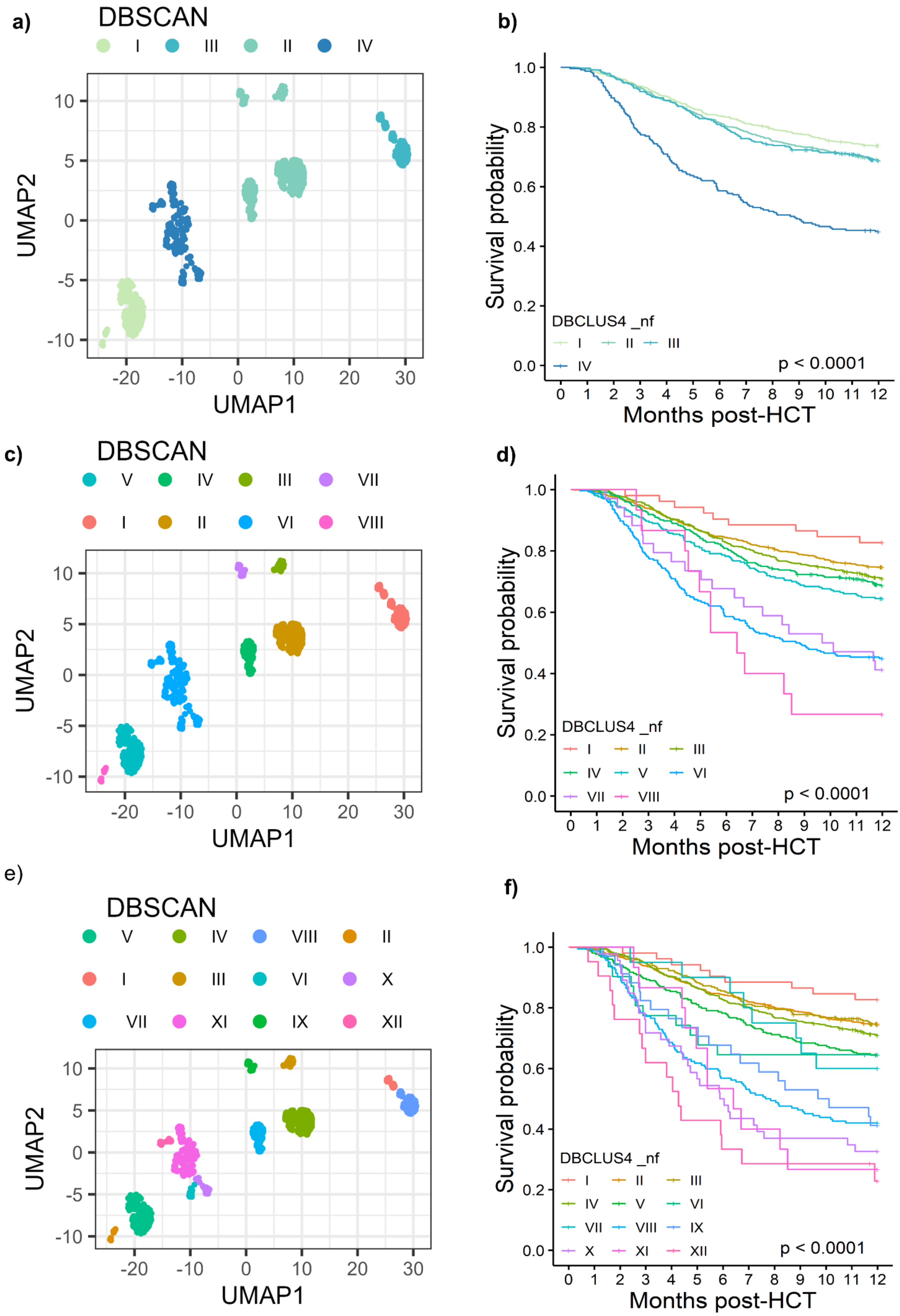

Supplementary Figure 5. Visualization of ascending DBSCAN cluster numbers on UMAP output and OS association, training cohort (n=2319)

Nonlinear dimensionality reduction with UMAP applied on the training cohort (n=2319) revealing two-dimensional data (UMAP1 and t-UMAP2) with each dot representing a single patient. The UMAP output was clustered using DBSCAN and patients colored according to the corresponding DBSCAN cluster. Explorative Kaplan-Meier analysis was performed to illustrate the spread of the clusters. **a** DBSCAN with 4 clusters. Colors representing DBSCAN cluster aGVHD severity grades I-IV (grade I, light green; grade II, dark green; grade III, teal, grade IV, blue) **b** Kaplan-Meier OS curve with 95% confidence interval (CI) of 4 clusters. Strata were compared with the two-sided log-rank test. **c** 8 clusters. Colors representing DBSCAN cluster aGVHD severity grades I-VIII. **d** Kaplan-Meier OS curve with 95% CI of 8 clusters. Strata were compared with the two-sided log-rank test. **e** 12 clusters. Colors representing DBSCAN cluster aGVHD severity grades I-XII. **f** Kaplan-Meier OS curve with 95% CI of 12 clusters. Strata were compared with the two-sided log-rank test.

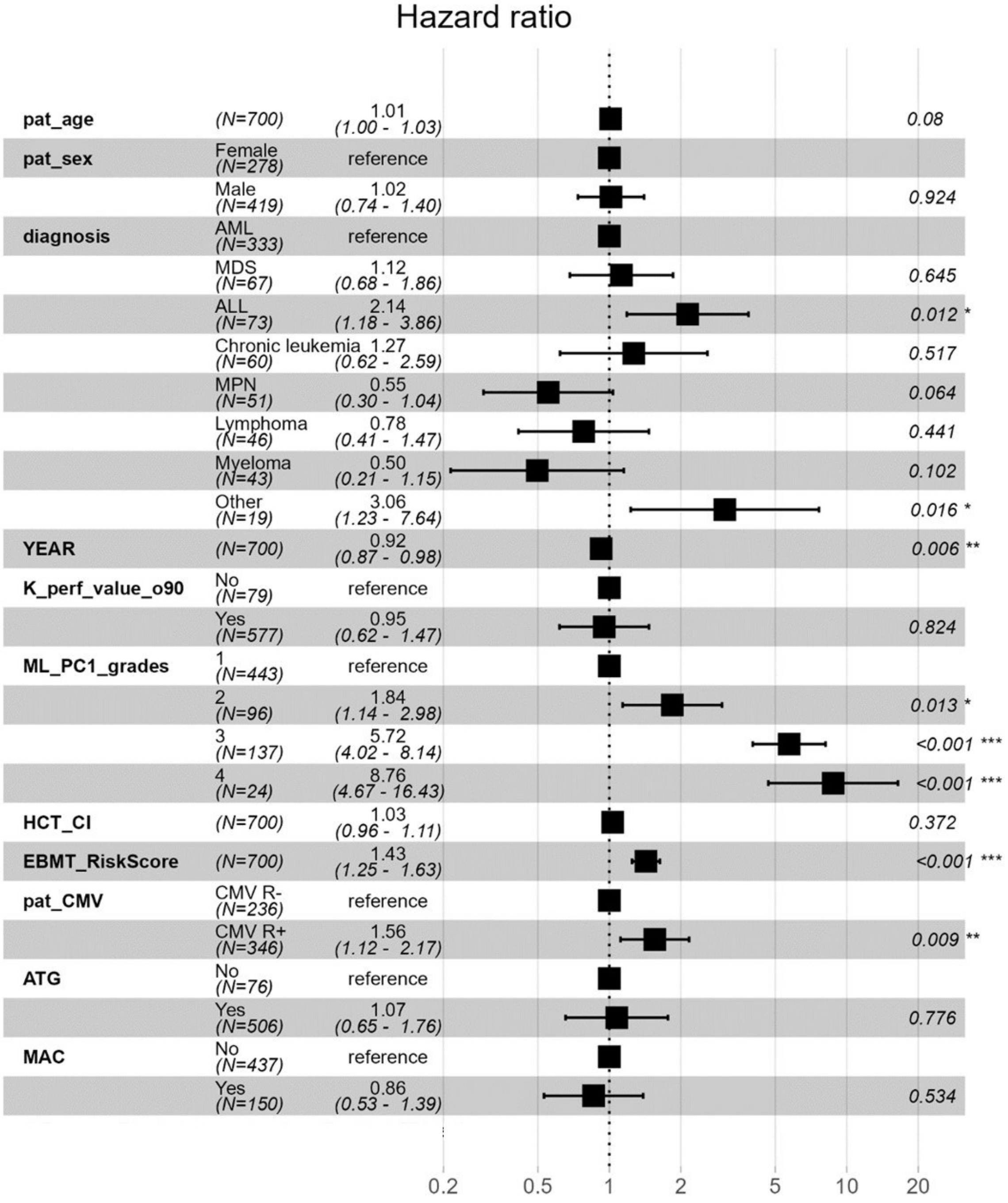

Supplementary Figure 6: Multivariate Cox regression analysis for 12 months OS using the PC1 aGVHD grades as time-dependent variable, test cohort (n=700) Other significant covariates for OS were some diagnoses (acute lymphoblastic leukemia (ALL), other diagnoses), year of HCT, EBMT risk score and cytomegalovirus positive serostatus of the recipient (CMV R+ serostatus). Age was no more significant once EBMT risk was included (which also

includes age). Horizontal bars represent 95% CI. P values were computed using the Wald test. The hazard ratio (HR) is a measure of the ratio of the hazard between two groups. A value of 1 is the reference. Source data are provided as a source data file.

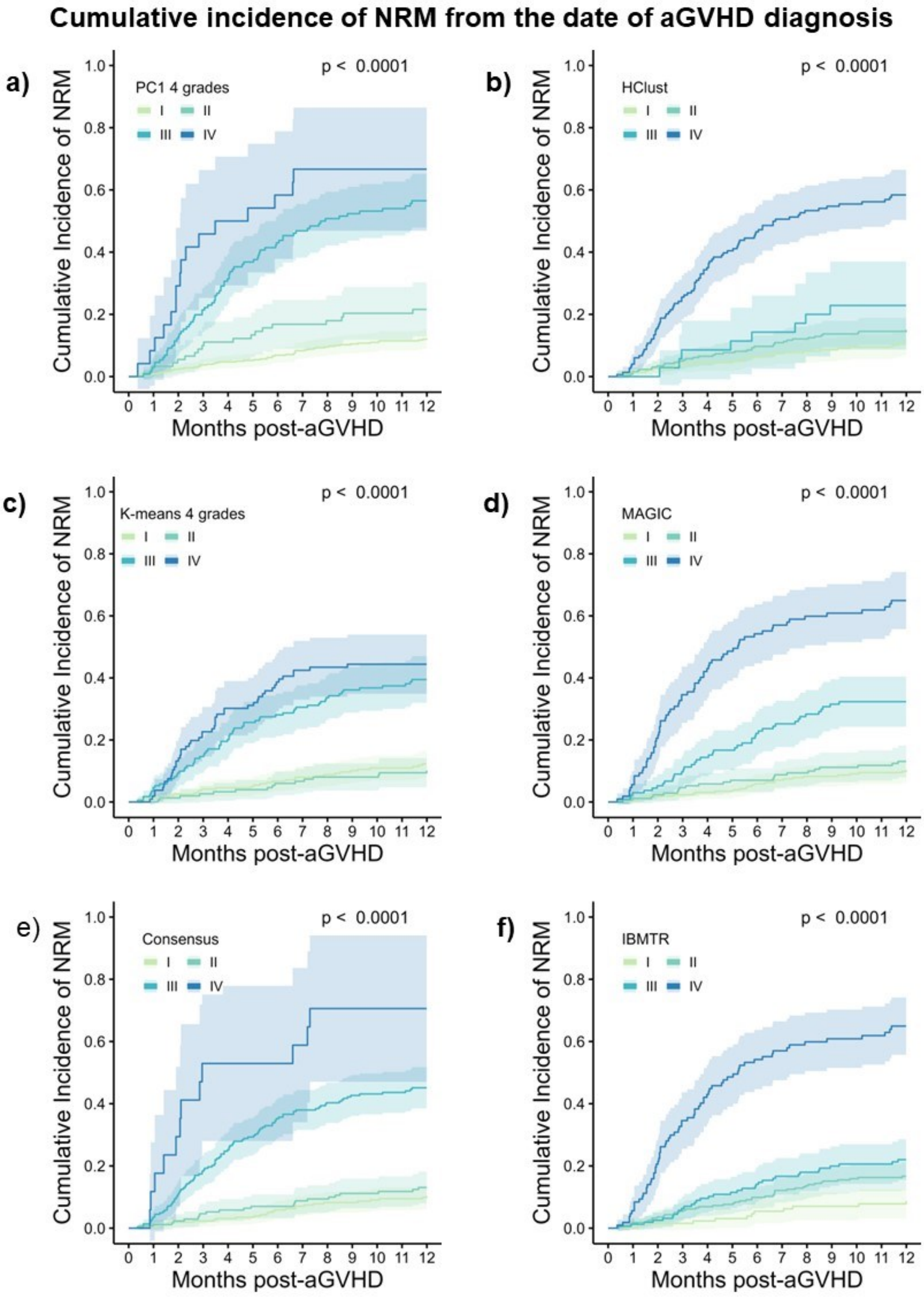

Supplementary Figure 7. Cumulative incidence of NRM from the date of aGVHD diagnosis. Test cohort (n=700). a PC1 with 4 grades. Colors representing PC1 aGVHD grades I-IV (I, light green; II, dark green; III, teal; IV, blue) **b** HClust grades I-IV **c** K-means 4 grades (grades I-IV) **d** MAGIC grades I-IV **e** Consensus grades I-IV **f** IBMTR grades I-IV. Error bands show 95% confidence interval. P values were computed using the two-sided Gray test.

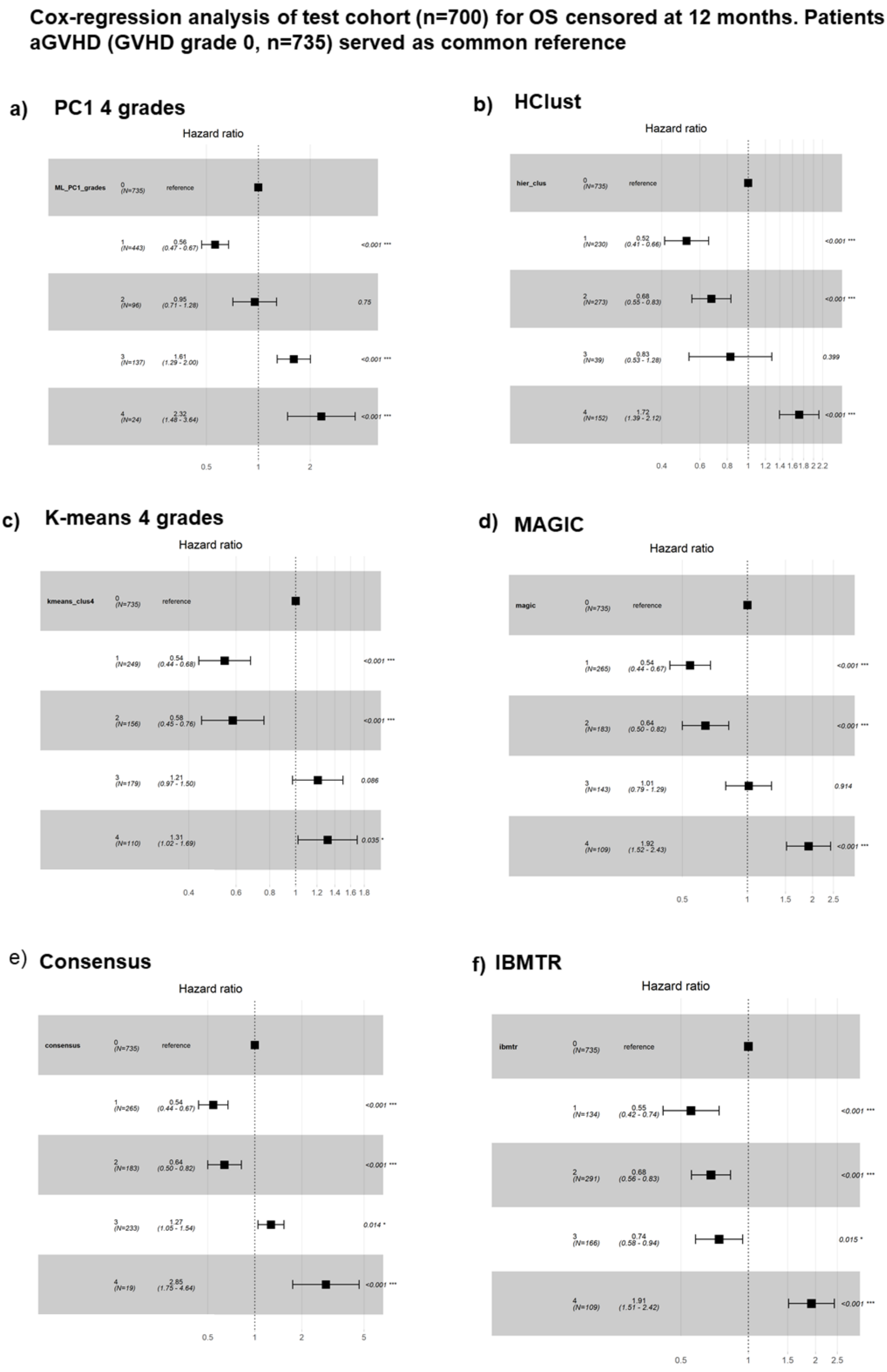

Supplementary Figure 8. Cox-regression analysis of test cohort (n=700) for OS censored at 12 months. Patients without aGVHD (GVHD grade 0, n=735) served as common reference

Illustrative comparison of hazard ratios (center boxes) between aGVHD classifications with 4

grades and a common reference cohort of patients with aGVHD grade 0 (same reference for both conventional and data-driven classifications). P values were computed using the Wald test. Source data are provided as a source data file. **a** PC1 4 grades **b** HClust **c** K-means 4 grades **d** MAGIC **e** Consensus **f** IBMTR. Error bars show 95% confidence interval.

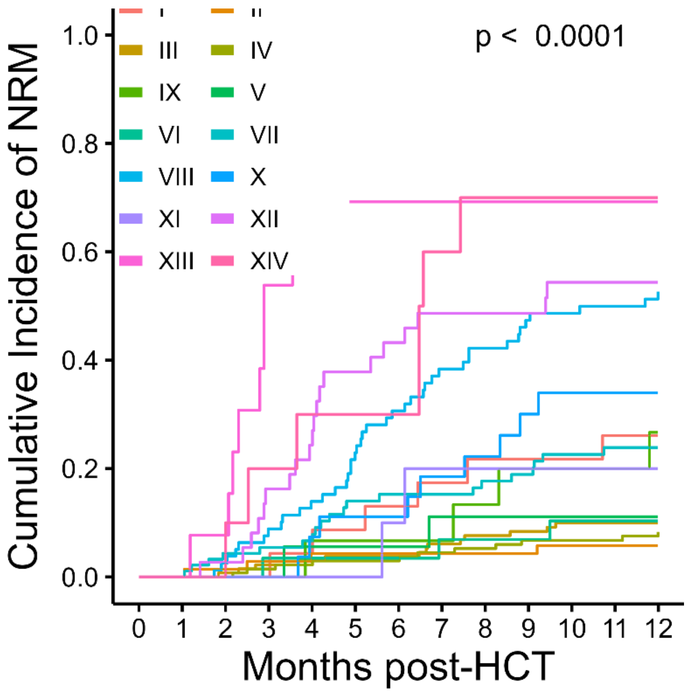

Supplementary Figure 9. Cumulative incidence of NRM of the K-means 14 clusters. Test cohort (n=700). Colors represent the different K-means aGVHD grades I-XIV. Strata are compared with the two-sided log-rank test.

Supplementary Tables

Supplementary Table 1: Baseline characteristics of training and test cohort

	**Training set**	**Validation set**	*p*
n	2319	700	
**Median age [IQR]**	55.00 [44.00, 62.00]	53.00 [43.00, 62.00]	0.680
**Male sex, n (%)**	1359 (58.6)	419 (59.9)	0.527
Missing	3 (0.1)	3 (0.4)	
**Diagnosis**			
AML	945 (40.8)	333 (47.6)	
MDS	335 (14.4)	67 (9.6)	
ALL	211 (9.1)	73 (10.4)	
Chronic leukemia	101 (4.4)	60 (8.6)	
MPN	248 (10.7)	51 (7.3)	
Lymphoma	251 (10.8)	46 (6.6)	
Myeloma	132 (5.7)	43 (6.1)	
Other	89 (3.8)	19 (2.7)	
Missing	7 (0.3)	8 (1.1)	
**Karnofsky at HCT**			<0.001
≥80	1796 (77.4)	635 (90.7)	
≥90	1162 (50.1)	577 (82.4)	
Missing	360 (15.5)	44 (6.3)	
**Female donor, n (%)**	775 (33.4)	195 (27.9)	0.752
Missing	3 (0.1)	131 (18.7)	
**Graft source, n (%)**			<0.001
PBSC	2106 (90.8)	561 (80.1)	
BM	207 (8.9)	25 (3.6)	
Missing	6 (0.3)	114 (16.3)	
**Donor type, n (%)**			
MRD	486 (21.0)	116 (16.6)	
MMRD (incl. haplo)	62 (2.7)	10 (1.4)	
Unrelated donor HCT	1683 (72.6)	461 (65.9)	
- MUD	533 (23.0)	335 (47.9)	
- Unrelated, not specified	899 (38.8)	0 (0.0)	
- MMUD	251 (10.8)	126 (18.0)	
Missing	88 (3.8)	113 (16.1)	
**CMV serostatus, n (%)**			
CMV D-/R-	668 (28.8)	189 (27.0)	0.013
CMV D-/R+	426 (18.4)	93 (13.3)	
CMV D+/R-	259 (11.2)	47 (6.7)	
CMV D+/R+	874 (37.7)	253 (36.1)	
Missing	92 (4.0)	118 (16.9)	
**RIC, n (%)**	734 (31.7)	437 (62.4)	<0.001
Missing	89 (3.8)	113 (16.1)	
**TBI, n (%)**	704 (30.4)	175 (25.0)	0.536
Missing	85 (3.7)	118 (16.9)	
**ATG (%)**	1582 (68.2)	506 (72.3)	<0.001
Missing	260 (11.2)	118 (16.9)	

Abbreviations: IQR, interquartile range; AML, acute myeloid leukemia; MDS, myelodysplastic syndromes; ALL, acute lymphoblastic leukemia; MPN, myeloproliferative neoplasm; PBSC, peripheral blood stem cells; BM, bone marrow; MRD, matched related donor; MMRD, mismatched related donor; haplo, haploidentical donor; MUD, matched unrelated donor; MMUD, mismatched unrelated donor;

CMV, cytomegatovirus; D, donor serostatus; R, recipient serostatus; RIC, reduced intensity conditioning; TBI, total body irradiation; ATG, anti-thymocyte globulin. P values were calculated using chi-square test; t-test was used for age.

Supplementary Table 2: Core outcomes of training and test cohort

	**Training set**	**Validation set**
**Acute GVHD**	2319 (100)	700 (100)
**PC1 grades, n (%)**		
PC1 grade I	1815 (78.3)	443 (63.3)
PC1 grade II	282 (12.2)	96 (13.7)
PC1 grade III	170 (7.3)	137 (19.6)
PC1 grade IV	52 (2.2)	24 (3.4)
**MAGIC grades, n (%)**		
MAGIC I	1018 (43.9)	265 (37.9)
MAGIC II	837 (36.1)	183 (26.1)
MAGIC III	314 (13.5)	143 (20.4)
MAGIC IV	150 (6.5)	109 (15.6)
**Chronic GVHD, n (%)**		
No	974 (42.0)	380 (54.3)
Yes	1344 (58.0)	257 (36.7)
Missing	1 (0.0)	63 (9.0)
**Relapse at 12 months, n (%)**	477 (20.6%)	103 (14.7%)
**Alive at 12 months, n (%)**	1576 (66.0%)	488 (69.7%)

Supplementary Methods

Non-linear data-driven approaches to the grading of aGVHD: Uniform Manifold Approximation and Projection (UMAP)[1, 2] and t-Distributed Stochastic Neighbor Embedding (tSNE)[3] were employed as previously described for visualizing different aGVHD grading systems in a 2-dimensional space. In short, tSNE uses a t-distribution curve to determine the similarity between all data points in the high-dimensional-space. The resulting similarity scores are stored in a matrix. tSNE then randomly plots the high dimensional data in 2 dimensions and again calculates a similarity matrix. Finally, the two similarity score matrices (of the high-dimensional and the 2-dimensional data) are compared within multiple iteration steps until both matrices are similar. Perplexity values between 1 and 751 in increments of 50 were tested, each with different iterations of 5, 10, 20, 50, 100, 200, 500 and 1000. We decided to use a perplexity of 51 with 10 iteration steps to represent aGVHD phenotypes in a 2D-plot. We also employed UMAP because it does not randomly plot the high dimensional data in 2 dimensions at initialization. Here again, low dimensional similarity scores based on a t-distribution curve are calculated and points are adjusted based on this score. We tested the n-neighbor values 2, 4, 16, 32, 64, 100 and 256, each with different minimum distances of 0.0125, 0.05, 0.2 and 0.8. DBSCAN is a density-based clustering that can be employed on UMAP output. The epsilon parameter specifies the distance between points to form clusters. For comparison with conventional grading, DBSCAN settings were configured to result in 4 clusters (corresponding to an epsilon set to 6)[4]. We further explored parameter settings on the output of UMAP, resulting in 5 to 13 clusters with epsilon set to 5.2, 5, 4, 2, 1.2, 1, 0.85, 0.79 and 0.78, respectively.

aGVHD phenotype composition: We comparatively analyzed the numbers and proportions of clinical aGVHD phenotypes (i.e. aGVHD organ-involvement compositions) in all data-driven aGVHD classifications as well as in the most relevant conventional grading system, MAGIC. Both absolute and relative contribution of phenotypes and overall heterogeneity were displayed using a multi-color scheme. The results are presented next to the respective

outcome association (e.g. Figure 6). Detailed list of the involved phenotypes are provided in the supplementary tables 3-13.

Analysis of redistributed patients: Some grading systems categorized a high number of aGVHD phenotypes in a single grade. Within these categories (e.g. in MAGIC grade III), we identified patients that were attributed to distinct aGVHD severity grades in other aGVHD grading systems, which we coined redistributed patients (e.g. MAGIC grade III patients that were classified by PC1 as grade II). These redistributed patients were analyzed separately from the remaining patients of the same severity grade and compared for differences in clinical outcome to dissect heterogeneity within selected large classification categories.

Description of institutional transplant protocols and practices: The studied patients received HCT for treatment of hematological malignancies. Donor search was performed by the respective transplant office at each institution in collaboration with donor-databases. Donors were HLA-matched related donors (MRD), mismatched-related and haploidentical related donors (MMRD, 10/10 HLA-A-, -B, -C, -DRB1, -DQB1 matched unrelated donors (MUD), or mismatched unrelated donors (MMUD). HLA-DPB1 was not considered for donor-recipient matching. The conditioning regimen was chosen by the treating physician considering diagnosis, age, comorbidities and donor constellation. Patients received the same early supportive and follow-up care. With the beginning of the conditioning regimen until discharge, all in patients were treated in reverse isolation single rooms with high-efficacy particle air filtration. Antiviral prophylaxis during neutropenia consisted of intravenous aciclovir at 250 mg three times daily. Antifungal prophylaxis consisted of oral posaconazole at 200 mg three times daily from day+1 with a minimal duration until day +100. Colony stimulating factors were not routinely applied. As pneumocystis-jirovecii pneumonia prophylaxis patients received either monthly pentamidin inhalation or oral cotrimoxazol at 960 mg three times per week from day +30. Only, irradiated red blood cell and platelet transfusions and in-line leukocyte-filtered products were used. Patients in both training and test cohorts received a pharmacological aGVHD prophylaxis including calcineurin inhibitors, most frequently in combination with either methotrexate or mycophenolate mofetil. Patients with higher GVHD risk (in particular those

with MUD and MMUD) were assigned to additional in vivo T cell depletion using anti-T-Lymphocyte globulin (ATG). In Essen, Heidelberg and Berlin, the preferred calcineurin inhibitor based GVHD prophylaxis was ciclosporin plus methotrexate (MTX), while in Hamburg and Hannover it was the combination of ciclosporin and mycophenolate mofetil (MMF). Ciclosporin was applied intravenously at 3 mg/kg bodyweight starting from day -1 before HCT e.g. in combination with 15 mg/m2 MTX on day +1 und 10 mg/m2 MTX on days +3, +6 and +11 after HCT. Normal early ciclosporin target blood levels (range, 150-250 ng/ml) were controlled three times weekly. MMF was started intravenously on day 0 at 30mg/kg bodyweight/day split into 2-3 daily doses. Following drug oralisation, MMF was continued orally depending on the protocol e.g. until day +28 for MRD/MUD or until day +35 (MMUD, MMRD). Polyvalent rabbit-ATG was applied at a dosage of 10 mg/kg bodyweight on days -4, -3 and -2 (cumulative dosage: ATG 30 mg/kg) or at a dosage of 20 mg/kg bodyweight on days -4, -3 and -2 (cumulative dosage: ATG 60 mg/kg). Before patient discharge, intravenous ciclosporin was substituted orally. Inpatients had daily medical visits until discharge around day+30. Outpatients were followed weekly until day+100.

Supplementary note 1: Non-linear data-driven approaches to the grading of aGVHD

In addition to the linear data science methods described in the main manuscript, we also explored common non-linear approaches on the data to address its heterogeneity, such as t-SNE (Suppl. Figure 3) and UMAP (Suppl. Figure 4). Both methods identified distinct patient clusters, illustrated as conflating dots, each dot representing one patient, and comparatively visualized the patient distributions in the data-driven and conventional grading systems. Additional, Density-Based Spatial Clustering of Applications with Noise (DBSCAN) using 4 grades following UMAP dimensionality reduction did not deliver satisfactory results, yet when dissecting the cohort into 8 to 12 DBSCAN clusters, its association with OS covered a broad spectrum of patient outcomes with distinct strata (Suppl. Figure 5). This approach holds potential for integrating biomarker features.

Supplementary note 2: Additional K-Means clustering with 14 clusters

Following the observation of a late plateau of the silhouette index on the development cohort at 14 K-means clusters (Figure 4C), we also explored a classification system leveraging these 14 K-means clusters on the independent test cohort (Suppl. Figure 9). Here, we found a slightly lower AIC of 2516 as well as a CI of 0.71, when validated on the test cohort. However, this system suffered from insufficiencies in the association with clinical outcome: 1) Numerous of the 14 severity grades were no distinct in OS or NRM, 2) the grades' severity did not align linearly in Cox regression with multiple jumps in the test cohort and 3) many of the cohorts were assembled only very few patients. We therefore did not further follow this approach.

Supplementary note 3: Employed R Libraries

ggplot2:

H. Wickham. ggplot2: Elegant Graphics for Data Analysis. Springer-Verlag New York, 2016.

readxl:

Hadley Wickham and Jennifer Bryan (2019). readxl: Read Excel Files. R package version 1.3.1. URL: https://CRAN.R-project.org/package=readxl

survival:

Terry M. Therneau, Patricia M. Grambsch (2000). _Modeling Survival Data: Extending the Cox Model_. Springer, New York. ISBN 0-387-98784-3.

survminer:

Alboukadel Kassambara, Marcin Kosinski and Przemyslaw Biecek (2021). survminer: Drawing Survival Curves using 'ggplot2'. R package version 0.4.9. URL: https://CRAN.R-project.org/package=survminer

cmprsk:

Bob Gray (2022). cmprsk: Subdistribution Analysis of Competing Risks. R package version 2.2-11. URL: https://CRAN.R-project.org/package=cmprsk

dplyr:

Hadley Wickham, Romain François, Lionel Henry and Kirill Müller (2022). dplyr: A Grammar of Data Manipulation. R package version 1.0.8. URL: https://CRAN.R-project.org/package=dplyr

ggstatsplot:

Patil, I. (2021). Visualizations with statistical details: The 'ggstatsplot' approach. Journal of Open Source Software, 6(61), 3167, doi:10.21105/joss.03167

dynpred:

Hein Putter (2015). dynpred: Companion Package to "Dynamic Prediction in Clinical Survival Analysis". R package version 0.1.2. URL: https://CRAN.R-project.org/package=dynpred

tidyr:

Hadley Wickham and Maximilian Girlich (2022). tidyr: Tidy Messy Data. R package version 1.2.0. URL: https://CRAN.R-project.org/package=tidyr

Rtsne:

L.J.P. van der Maaten and G.E. Hinton. Visualizing High-Dimensional Data Using t-SNE. Journal of Machine Learning Research 9(Nov):2579-2605, 2008.

L.J.P. van der Maaten. Accelerating t-SNE using Tree-Based Algorithms. Journal of Machine Learning Research 15(Oct):3221-3245, 2014.

Jesse H. Krijthe (2015). Rtsne: T-Distributed Stochastic Neighbor Embedding using a Barnes-Hut Implementation, URL: https://github.com/jkrijthe/Rtsne

umap:

Tomasz Konopka (2022). umap: Uniform Manifold Approximation and Projection. R package version 0.2.8.0. URL: https://CRAN.R-project.org/package=umap

tidyverse:

Wickham et al., (2019). Welcome to the tidyverse. Journal of Open Source Software, 4(43), 1686, URL: https://doi.org/10.21105/joss.01686

writexl:

Jeroen Ooms (2021). writexl: Export Data Frames to Excel 'xlsx' Format. R package version 1.4.0. URL: https://CRAN.R-project.org/package=writexl

aod:

Lesnoff, M., Lancelot, R. (2012). aod: Analysis of Overdispersed Data. R package version 1.3.1, URL: http://cran.r-project.org/package=aod

tableone:

Kazuki Yoshida and Alexander Bartel (2022). tableone: Create 'Table 1' to Describe Baseline Characteristics with or without Propensity Score Weights. R package version 0.13.2. URL: https://CRAN.R-project.org/package=tableone

timeROC:

Paul Blanche, Jean-Francois Dartigues, Helene Jacqmin-Gadda (2013). Estimating and Comparing time-dependent areas under receiver operating characteristic curves for censored event times with competing risks. Statistics in Medicine, 32(30), 5381-5397. URL: http://onlinelibrary.wiley.com/doi/10.1002/sim.5958/full

Supplementary note 4: Employed Python libraries

scikit-learn

Pedregosa *et al.*, Scikit-learn: Machine Learning in Python, JMLR 12, pp. 2825-2830, 2011.

numpy

Harris, C.R., Millman, K.J., van der Walt, S.J. et al. *Array programming with NumPy*. Nature 585, 357–362 (2020). DOI: 10.1038/s41586-020-2649-2.

scipy

Pauli Virtanen, Ralf Gommers, Travis E. Oliphant et al.. (2020) SciPy 1.0: Fundamental Algorithms for Scientific Computing in Python**.** *Nature Methods*, 17(3), 261-272.

pandas

McKinney W, others. Data structures for statistical computing in python. In: Proceedings of the 9th Python in Science Conference. 2010. p. 51–6.

Matplotlib

J. D. Hunter, "Matplotlib: A 2D Graphics Environment", Computing in Science & Engineering, vol. 9, no. 3, pp. 90-95, 2007.

seaborn

Waskom ML. seaborn: statistical data visualization. Journal of Open Source Software. 2021;6(60):3021. doi: 10.21105/joss.03021.

PyYAML

LibYAML, release 0.2.5, 2020-06-01 URL: https://github.com/yaml/pyyaml.org or https://pyyaml.org/

Supplementary References

1. Becht E*, et al.* Dimensionality reduction for visualizing single-cell data using UMAP. *Nat Biotechnol*, (2018).

2. MCInnes L, Healy, J., Melville, J. UMAP: Uniform Manifold Approximation and Projection for Dimension Reduction. (2018).

3. Maaten LVd. Visualizing data using t-SNE. *Journal of Machine Learning Research* **9**, 2579-2605 (2008).

4. Ester M, Kriegel H-P, Sander J, Xu X. A density-based algorithm for discovering clusters in large spatial databases with noise. In: *Proceedings of the Second International Conference on Knowledge Discovery and Data Mining*). AAAI Press (1996).
