# Supplementary material for: Data-driven grading of acute graft-versus-host disease
Source: Nat Commun. 2023 Nov 28;14:7799. doi: 10.1038/s41467-023-43372-2 (PMC10684603; doi:10.1038/s41467-023-43372-2)
Supplement: Supplementary file 3 — Description of Additional Supplementary Files [file 41467_2023_43372_MOESM3_ESM.pdf]

Description of Additional Supplementary Files

File name: Supplementary Data 1

Description: Proportion of patients in the external test cohort with their respective aGVHD phenotype per MAGIC Grades I-IV.

File name: Supplementary Data 2

Description: Proportion of patients in the external test cohort with their respective aGVHD phenotype per PC1 Grades I-IV.

File name: Supplementary Data 3

Description: Proportion of patients in the external test cohort with their respective aGVHD phenotype per Hierarchical clustering Grades I-IV.

File name: Supplementary Data 4

Description: Proportion of patients in the external test cohort with their respective aGVHD phenotype per K-means clustering Grades I-IV.

File name: Supplementary Data 5

Description: Proportion of patients in the external test cohort with their respective aGVHD phenotype per Consensus Grades I-IV.

File name: Supplementary Data 6

Description: Proportion of patients in the external test cohort with their respective aGVHD phenotype per IBMTR Grades I-IV.

File name: Supplementary Data 7

Description: Proportion of patients in the external test cohort with their respective aGVHD phenotype per Minnesota Grades I-IV.

File name: Supplementary Data 8

Description: Proportion of redistributed MAGIC grade III patients in the external test cohort within PC1 grades I and II including the aGVHD phenotype.

File name: Supplementary Data 9

Description: Proportion of patients in the external test cohort with their respective aGVHD phenotype per PC1 Grades I-XII.

File name: Supplementary Data 10

Description: Proportion of patients in the external test cohort with their respective aGVHD phenotype per PC1 Grades I-VI.

File name: Supplementary Data 11

Description: Proportion of patients in the external test cohort with their respective aGVHD phenotype per K-means Grades I-VIII.
